# Can Synthetic Data Allow for Smaller Sample Sizes in Chronic Urticaria Research?

**DOI:** 10.1002/clt2.70087

**Published:** 2025-08-07

**Authors:** Annika Gutsche, Pascale Salameh, Samad S. Jahandideh, Mehran Roodsaz, Serkan Kutan, Ali Salehzadeh‐Yazdi, Emek Kocatürk, Stamatios Gregoriou, Simon F. Thomsen, Kanokvalai Kulthanan, Papapit Tuchinda, Joachim Dissemond, Alicja Kasperska‐Zajac, Magdalena Zajac, Mateusz Zamłyński, Martijn van Doorn, Claudio A. S. Parisi, Jonny G. Peter, Cascia Day, Cathryn McDougall, Michael Makris, Daria Fomina, Elena Kovalkova, Nikolai Streliaev, Gerelma Andrenova, Marina Lebedkina, Maryam Khoskhkui, Mehraneh M. Aliabadi, Andrea Bauer, Lea Kiefer, Melba Muñoz, Karsten Weller, Pavel Kolkhir, Martin Metz

**Affiliations:** ^1^ Institute of Allergology Charité—Universitätsmedizin Berlin Corporate Member of Freie Universität Berlin and Humboldt‐Universität zu Berlin Berlin Germany; ^2^ Fraunhofer Institute for Translational Medicine and Pharmacology ITMP, Immunology and Allergology Berlin Germany; ^3^ Faculty of Pharmacy Lebanese University Beirut Lebanon; ^4^ Gilbert and Rose–Marie Chagoury School of Medicine Lebanese American University Beirut Lebanon; ^5^ Department of Primary Care and Population Health University of Nicosia Medical School Nicosia Cyprus; ^6^ Institut National de Santé Publique d’Épidémiologie Clinique et de Toxicologie‐Liban (INSPECT‐LB) Beirut Lebanon; ^7^ Tediax B.V. Sterrenbos 5 Valburg the Netherlands; ^8^ School of Science Constructor University Bremen Germany; ^9^ Department of Dermatology Bahçeşehir University School of Medicine Istanbul Turkiye; ^10^ Faculty of Medicine National and Kapodistrian University of Athens Andreas Sygros Hospital Athens Greece; ^11^ Department of Dermatology Bispebjerg Hospital University of Copenhagen Biomedical Sciences Copenhagen Denmark; ^12^ Department of Dermatology Faculty of Medicine Siriraj Hospital Mahidol University Bangkok Nakhon Pathom Thailand; ^13^ Department of Dermatology, Venerology and Allergology University of Essen Essen Germany; ^14^ Department of Clinical Allergology and Urticaria of Medical University of Silesia Katowice Poland; ^15^ Department of Dermatology Urticaria Center of Reference and Excellence Erasmus MC Rotterdam the Netherlands; ^16^ Adults and Pediatric Allergy Sections Italian Hospital of Buenos Aires Buenos Aires Argentina; ^17^ Allergy and Immunology Unit University of Cape Town Lung Institute Cape Town South Africa; ^18^ Allergy Unit 2nd Dpt. Dermatology and Venereology National and Kapodistrian University of Athens University General Hospital “Attikon” Athens Greece; ^19^ Moscow Research and Practical Center of Allergy and Immunology Moscow Healthcare Department City Clinical Hospital Moscow Russian Federation; ^20^ Department of Clinical Immunology and Allergology I.M. Sechenov First Moscow State Medical University (Sechenov University) Moscow Russian Federation; ^21^ Department of Pulmonology Astana Medical University Astana Kazakhstan; ^22^ Allergy Research Center Mashhad University of Medical Sciences Mashhad Iran; ^23^ Department of Dermatology University Hospital Carl Gustav Carus Technical University Dresden Dresden Germany

**Keywords:** chronic spontaneous urticaria (CSU), real‐world data (RWD), sensitivity analysis, subgroup analysis, synthetic data generation, tree‐based decision model

## Abstract

**Background:**

Robust data are essential for clinical and epidemiological research, yet in chronic spontaneous urticaria (CSU), certain patient groups, such as the elderly or comorbid patients, are often underrepresented. In clinical trials, strict inclusion and exclusion criteria frequently limit recruitment, making it difficult to achieve sufficient statistical power. Similarly, real‐world observational studies may lack sufficient sample sizes for robust analysis. To address these limitations, we generated synthetic patient data that reflect these groups’ clinical characteristics and variability. This approach enables more comprehensive analyses, facilitates hypothesis testing in otherwise inaccessible populations, and supports the generation of evidence where traditional data sources are insufficient.

**Methods:**

A tree‐based decision model was applied to generate synthetic data based on an existing set of real‐world data (RWD) from the Chronic Urticaria Registry (CURE). Descriptive characteristics and association strength between relevant RWD variables and their synthetic counterparts were analyzed as indicators of replication accuracy, providing insight into how closely the synthetic data aligns with the RWD. Finally, we determined the minimum sample size required to generate high‐quality synthetic data.

**Results:**

The algorithm produced extensive synthetic data records, closely mirroring patient demographics and disease clinical characteristics. Smaller subgroups of the data were equally replicated and followed the same distribution as RWD. Known associations and correlations between disease‐specific factors (disease control) and risk factors (age) yielded similar results, with no significant difference (*p* > 0.05). The lowest threshold at which synthetic data could be generated while maintaining high accuracy in RWD was identified to be 25%, enabling a fourfold increase in the synthetic population.

**Conclusion:**

Synthetic data could replicate RWD with reasonable accuracy for patients with CSU down to 25% of the original population size. This method has the potential to extend small patient subgroups in clinical and epidemiological research.

## 1 Introduction

Randomized controlled trials (RCTs) remain the gold standard in evidence‐based medicine, yet conducting large clinical trials is challenging due to the high costs and resources they demand. Commonly, patients with various chronic diseases, such as chronic spontaneous urticaria (CSU), are chosen based on strict inclusion and exclusion criteria [1]. CSU is a skin condition marked by the recurrence of itchy wheals (hives), angioedema, or both for more than 6 weeks [2]. Despite the moderately high prevalence of CSU (0.5%–1% in the general population), RCTs often face significant challenges in recruiting and retaining patients [3]. In particular, specific subgroups of patients with CSU, such as those with relevant comorbidities [4], the elderly [5], those with different skin types [6], non‐responders to previous treatments, or patients with specific underlying pathogenic pathways [7], are often understudied. Furthermore, scientists and researchers face limited patient numbers, a high number of screening failures due to comorbidities, or drop outs [8], which prompts them to seek novel methods of analysis [9].

Synthetic data derived from validated real‐world data (RWD) can be used to simulate trial arms, conduct subgroup sensitivity analyses, or augment statistical models, especially in trials where limited patient data are available [10, 11, 12]. Studies have shown that synthetic data can replace real data values with simulated ones [9, 13, 14]. By training interpretable generative models on these RWD sources, synthetic patient cohorts can be created that replicate clinically relevant distributions and associations [15, 16]. While synthetic data is not a replacement for RCTs, it offers many advantages: data is scalable, privacy is preserved, and relevance regarding smaller patient groups is extended, provided the underlying models are transparent and validated against real‐world benchmarks. Additionally, synthetic data can be generated from pseudonymized data; once synthesized, the data possesses the characteristics of fully anonymized data, which reduces the risks associated with sharing sensitive patient health information with third parties. Taken together, synthetic data can potentially increase data accessibility, enhance privacy protection, and address biases present in original datasets [5, 17].

However, researchers must be aware of the limitations, such as the risk of data leakage, dependency on imputation models, and the potential to exacerbate existing biases. Furthermore, synthetic data resilience, particularly regarding privacy and bias awareness, might depend on the field and type of data involved [5, 18]. Many key areas remain uncertain, including the selection of fit‐for‐purpose input data, the choice of an appropriate model, the evaluation of model performance, and the interpretation of results.

Our goal in the current study is to assess the application of synthetic data generation to support small samples resulting from poor recruitment and retention in CSU clinical trials. We aim to evaluate whether a generative model can produce synthetic datasets that closely mirror patient characteristics, accurately replicate baseline data, patient subgroups, key associations (e.g., age and disease duration), and identify the minimum sample size of real‐world data required to generate high‐quality synthetic data.

## 2 Methods

### 2.1 Patient Population

This study used original data from Chronic Urticaria Registry (CURE). CURE is a prospective, international, multicentre registry. It collects baseline and follow‐up data (every 6 months) using physician and patient questionnaires. This study included data from CURE from its initiation to April 2023, with extracted baseline records of 4136 patients aged ≥ 2 years with physician‐verified CSU. Data were collected from 54 CURE centers representing 30 countries, including 12 ethnicities.

We analyzed 19 variables, covering demographic characteristics, laboratory results, and patient‐reported outcome measurements (PROMs) such as the Urticaria Activity Score over 7 days (UAS7) and the Urticaria Control Test (UCT). The total sample size varies across different variables due to differences in data availability and missing values. Additionally, the baseline (starting time point) and disease duration differed for each patient.

### 2.2 Ethical Approval

CURE was approved in 2014 by the Ethics Committee of the Charité—Universitätsmedizin Berlin, Germany (reference EA1/146/14). All participating centers locally obtained ethics committee approvals, and all participants gave a written informed consent before joining the registry.

### 2.3 Generative Model

Unstructured patient data were de‐identified and converted to structured data using a generative Decision Tree (GenDT), followed by extraction of potential predictive variables at the patient level encompassing demographic and disease‐specific characteristics. We then employed GenDT to create synthetic datasets from the actual RWD patient data. Characteristics of the generated synthetic data, including the distribution of demographic and clinical variables, were then examined and compared.

Registry data involves unstructured data, missingness, noise, or unknown interactions, therefore, we used the Classification and Regression Trees (CART) algorithm from the synthpop package in R, which can handle large and complex datasets [2, 13, 19].

**FIGURE 1:**
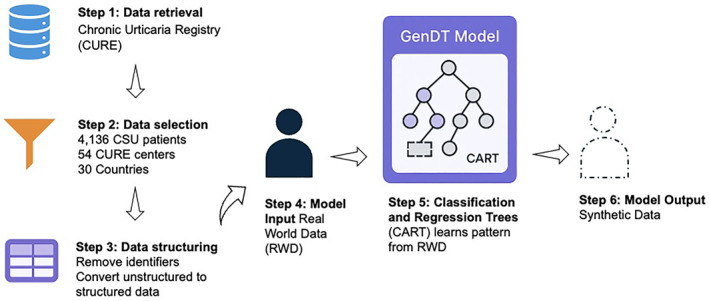
This flowchart illustrates the process of generating synthetic patient data using a Generative Decision Tree (GenDT) model. It begins with retrieving data followed by selecting data based on inclusion criteria and finally structuring the data to feed into the model. The model then applies the CART algorithm to learn underlying patterns and relationships. The resulting synthetic data mimics the statistical properties, distributions, and correlations of the original dataset.

One of its key advantages is its versatility, as it effectively handles categorical and numerical features across classification and regression tasks. CART also accommodates missing values and outliers, reducing the need for extensive data preprocessing [20, 21]. It models the relationships and distributions within the original dataset, using the generated models to create new, artificial data points that maintain the statistical properties and correlations of the original data without directly copying any individual records. This approach effectively anonymizes the data while retaining its utility for analysis [13, 22]. This allows CART to remain interpretable while capturing key clinical patterns in the data (Figure 1).

We assessed the quality of the synthetic data using established similarity metrics (pMSE and standardized pMSE), which compare how well the synthetic data replicate the structure of the original patient data. Lower values on these metrics suggest higher similarity.

### 2.4 Statistical Analysis

Continuous data was described by mean, standard deviation, median, and interquartile range, while multinomial data was presented as frequency and percentage. Associations were assessed using Pearson or Spearman correlation coefficients, beta coefficients from simple regressions, or odds ratios for dichotomous variables in respective datasets. When necessary, 95% Confidence intervals (CIs) were also presented. We then compared parameters, model coefficients, and CIs between the analyses conducted on the original and synthetic datasets. We used visualization techniques to compare univariate and bivariate distributions between the two datasets. A high degree of overlap in these measures would indicate that the synthetic data can produce valid inferences like those obtained from the original data. Analyses were also conducted over patient subgroups, including age (elderly > 60 years), gender, body mass index (BMI), number of comorbidities, UAS7 and UCT scores. Multivariable analyses included an interaction term between the independent variable and data type to test for significant differences in their associations with the dependent variable. Model coefficients and CIs from analyses of the original and synthetic datasets were compared. The distribution of *p*‐values from RWD was estimated and compared with those from synthetic data to assess the likelihood of Type I errors. Bootstrapping was applied to estimate the distribution of a test statistic by repeatedly sampling with replacement from RWD and synthetic data.

Furthermore, to define the minimum number of patients' records necessary for generating high‐quality synthetic data, we calculated the sample size for 95% targeted power and randomly selected a subset of the entire dataset. Afterward, we used a range of randomly selected portions of data to create synthetic data with the same size of the calculated sample size and compared the real data at variable levels.

All data cleaning, mining, and modeling steps were performed using the R statistical computing system (version 4.3.3; R Core Team, 2016), the R base packages, and the add‐on R packages synthpop (version 1.8.0) [23], rpart (version 4.1.23) [19], tree (version 1.0.43) [24], ggplot2 (version 3.5.1) [25], emmeans (version 1.10.3) [26], and dplyr (version 1.1.4) [25].

## 3 Results

### 3.1 Synthetic Data Closely Mirrors the Patient Demographics of RWD

**FIGURE 2:**
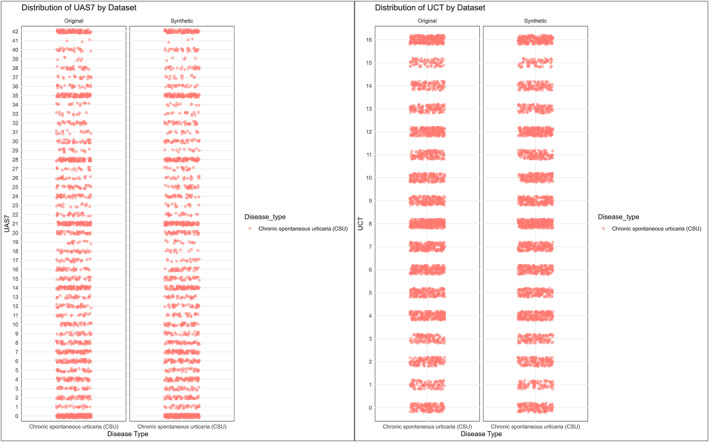
Comparison of UAS7 (left) and UCT (right) score distributions between original (real‐world) and synthetic datasets for chronic spontaneous urticaria (CSU) patients. Red dots represent individual patient scores, highlighting similar distributions across both datasets.

For all patient‐level characteristics (gender, age, BMI, disease type, ethnicity, etc.), we observed an accurate reflection of the RWD distribution (Table 1). For example, in terms of gender, the RWD reports 72.4% female (*n* = 2994) with a 95% CI (71.0%, 73.8%), while the synthetic data indicates 71.7% female (*n* = 2965) with a 95% CI (70.3%, 73.1%) with a *p*‐value demonstrating no significant difference. For males, the RWD reports a percentage of 28 (*n* = 1142) with a 95% CI (26.2%, 29.0%), while the synthetic data shows 28% (*n* = 1171) with a 95% CI (26.9%, 29.7%), also indicating close alignment. In terms of age distribution, the RWD provides a mean age of 44 years (SD = 16.3) with a 95% CI (43.7, 44.7), compared to 44 years (SD = 16.4) with a 95% CI (43.8, 44.8) in the synthetic data (Figure 2). The high *p*‐value of 0.9 further indicates an accurate replication of the age distribution between the real and synthetic datasets.

**TABLE 1:** Baseline characteristics of patients comparing real‐world and synthetic data across demographics and clinical parameters.

	Real‐world data (*n*(%); [95% CI])	Synthetic data (*n*(%); [95% CI])	*p*‐value
Gender			
Female	2994 (72.4%); [71.0 to 73.8]	2965 (71.7%); [70.3 to 73.1]	0.47
Male	1142 (27.6%); [26.2 to 29.0]	1171 (28.3%); [26.9 to 29.7]	
Continuous characteristic			
Age	44.2 (16.3); [43.73 to 44.72]	44.3 (16.4); [43.79 to 44.79]	0.85
BMI	26.26 (6.69); [26.04 to 26.49]	26.11 (5.52); [25.92 to 26.29]	0.28
Number of comorbidities	1.98 (1.75); [1.92 to 2.03]	1.96 (1.70); [1.91 to 2.02]	0.77
Lab values (IgE, etc.)	182.35 (292.17); [163.42 to 201.27]	185.75 (294.86); [166.85 to 204.65]	0.80
Medication taken			
2^nd^ gen. AH high dose	1123 (27.15%); [25.8 to 28.51]	1112 (26.89%); [25.53 to 28.24]	0.99
2^nd^ gen. AH stand. dose	1364 (32.98%); [31.55 to 34.41]	1351 (32.66%); [31.24 to 34.09]	
Cyclosporin	24 (0.58%); [0.35 to 0.81]	25 (0.6%); [0.37 to 0.84]	
No treatment	559 (13.52%); [12.47 to 14.56]	562 (13.59%); [12.54 to 14.63]	
Omalizumab high dose	45 (1.09%); [0.77 to 1.4]	50 (1.21%); [0.88 to 1.54]	
Other	559 (13.52%); [12.47 to 14.56]	571 (13.81%); [12.75 to 14.86]	
Type of symptoms (episodic vs. permanent)			
No	2018 (48.79%); [47.27 to 50.31]	2060 (49.81%); [48.28 to 51.33]	0.30
Yes	1850 (44.73%); [43.21 to 46.24]	1801 (43.54%); [42.03 to 45.06]	
Not reported	268 (6.48%); [5.73 to 7.23]	275 (6.65%); [5.89 to 7.41]	
Frequency of wheals			
0 days	472 (11.41%); [10.44 to 12.38]	469 (11.34%); [10.37 to 12.31]	0.98
1–3 days	548 (13.25%); [12.22 to 14.28]	566 (13.68%); [12.64 to 14.73]	
4–6 days	285 (6.89%); [6.12 to 7.66]	299 (7.23%); [6.44 to 8.02]	
7–13 days	327 (7.91%); [7.08 to 8.73]	320 (7.74%); [6.92 to 8.55]	
every day	1181 (28.55%); [27.18 to 29.93]	1192 (28.82%); [27.44 to 30.2]	
Not reported	562 (13.59%); [12.54 to 14.63]	548 (13.25%); [12.22 to 14.28]	
Frequency of angioedema			
0 days	1006 (24.32%); [23.02 to 25.63]	982 (23.74%); [22.45 to 25.04]	0.85
1 day	385 (9.31%); [8.42 to 10.19]	400 (9.67%); [8.77 to 10.57]	
2–3 days	524 (12.67%); [11.66 to 13.68]	496 (11.99%); [11.0 to 12.98]	
4–6 days	273 (6.6%); [5.84 to 7.36]	264 (6.38%); [5.64 to 7.13]	
Not reported	1691 (40.88%); [38.64 to 43.14]	1739 (42.05%); [39.77 to 44.32]	
Ethnicity			
African	32 (0.77%); [0.51 to 1.04]	27 (0.65%); [0.41 to 0.9]	0.78
Caribbean	2 (0.05%); [−0.02 to 0.12]	3 (0.07%); [−0.01 to 0.15]	
Caucasian	2701 (65.3%); [63.85 to 66.76]	2746 (66.39%); [64.95 to 67.83]	
Caucasian, ethnicity unknown	4 (0.1%); [0 to 0.19]	2 (0.05%); [−0.02 to 0.12]	
Caucasian, Latino/Hispanic	3 (0.07%); [−0.01 to 0.15]	2 (0.05%); [−0.02 to 0.12]	
East Asian	137 (3.31%); [2.77 to 3.86]	136 (3.29%); [2.74 to 3.83]	
Ethnicity unknown	426 (10.3%); [9.37 to 11.23]	414 (10.01%); [9.09 to 10.92]	
Latino/Hispanic	154 (3.72%); [3.15 to 4.3]	156 (3.77%); [3.19 to 4.35]	
Middle east	375 (9.07%); [8.19 to 9.94]	327 (7.91%); [7.08 to 8.73]	
South Asian	24 (0.58%); [0.35 to 0.81]	21 (0.51%); [0.29 to 0.72]	
Not reported	274 (6.62%); [5.87 to 7.38]	297 (7.18%); [6.39 to 7.97]	
Substance abuse			
Tobacco	328 (7.93%); [7.11 to 8.75]	348 (8.41%); [7.57 to 9.26]	0.44
Not reported (tobacco)	3808 (92.1%); [91.2 to 92.9]	3788 (91.6%); [90.7 to 92.4]	
Cannabis	9 (0.22%); [0.08 to 0.36]	6 (0.15%); [0.03 to 0.27]	0.44
Not reported (cannabis)	4127 (99.8%); [99.6 to 99.9]	4130 (99.9%); [99.7 to 100]	

*Note:* An ethnicity with fewer than two representatives were removed for clarity.

Abbreviations: % = Percentage, 2nd gen. AH = Second‐generation antihistamines, BMI = Body Mass Index, CI = Confidence Interval, IgE = Immunoglobulin E, *n* = number of samples, stand. dose = Standard dose.

### 3.2 Synthetic Data Closely Mirrors the Clinical Disease Characteristics of RWD

The frequency of symptoms per week was consistent across datasets (Table 1). For example, symptoms occurring daily were reported in 32.4% of RWD and 32.7% of synthetic data, while < 1 day was noted in 16.2% and 15.6%, respectively. Similarly, the frequency of wheals and angioedema was closely aligned between datasets. Daily wheals occurred in 28.6% of RWD and 28.8% of synthetic data, while no wheals were reported for 11.4% and 11.3%, respectively. Angioedema was reported by 6.6% of the RWD population every 4–6 days, while 6.4% of the synthetic population was affected. Similarly, 24.3% of CSU patients in the RWD reported no angioedema, which did not differ significantly from the 23.7% reported in the synthetic data (Table 1).

As shown in Table 1, the number of comorbidities was similar between RWD and synthetic data, with means of 1.98 (95% CI: [1.92–2]) and 1.96 (95% CI: [1.9–2]), respectively (*p* = 0.8). When examined by specific comorbidities, no significant differences were observed between RWD and synthetic data. For example, atopic dermatitis was reported in 4.8% of patients in RWD compared to 4.8% in synthetic data (*p* = 0.95), and allergic rhinitis was found in 19.1% and 19.2% of patients, respectively (*p* = 0.98; Table A1). Medication usage was also comparable, with no significant differences observed across all categories, including using high‐dose second‐generation antihistamines (27.2% vs. 26.9%, *p* = 0.99) and no treatment (13.5% vs. 13.6%, *p* = 0.99).

**FIGURE 3:**
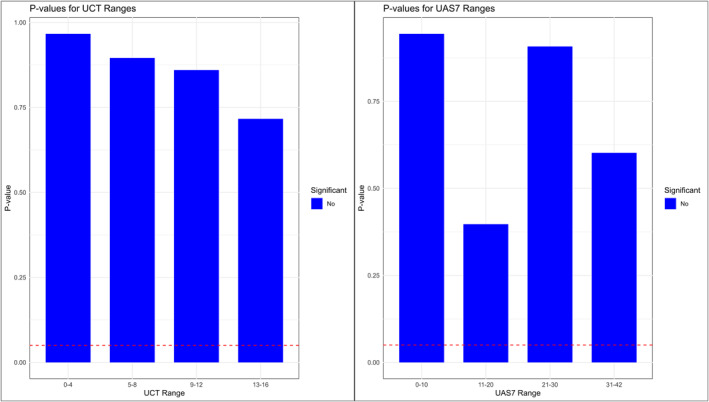
Comparison of UCT (left) and UAS7 (right) score ranges between original and synthetic datasets shows no statistically significant differences across all ranges. Blue bars represent the *p*‐values for each score range, with the red dashed line indicating the significance threshold (*p* = 0.05). All *p*‐values exceed the threshold, confirming similarity between the datasets.

PROMs, including UCT and UAS7, were replicated in synthetic data, with identical medians for UCT (8 [7] in RWD vs. 8 [8] in synthetic, *p* = 0.58) and UAS7 (15 [23] in RWD vs. 14 [24] in synthetic, *p* = 0.18). The analysis demonstrated identical score ranges between RWD and synthetic data (Table 2). For the UCT score, all intervals (0–4, 5–8, 9–12, 13–16) had *p*‐values > 0.7, indicating no significant differences. Similarly, for the UAS7 score, all intervals (0–10, 11–20, 21–30, 31–42) had *p*‐values > 0.3, further confirming consistency between the datasets (Figure 3).

**TABLE 2:** Comparison between real world and synthetic data on patient reported outcome measures (PROMs).

Ordinal/continuous	Category	Real world median [IQR]/*n*, (%), 95% CI	Synthetic median [IQR]/*n*, (%), 95% CI	*p* value
Urticaria control test (UCT)		8 [7]	8 [8]	0.58
Urticaria activity score (UAS7)		15 [23]	14 [24]	0.18
Physician global assessment (PGA)	Mild	1367, 33.05%, 31.62–34.48	1400, 33.85%, 32.41–35.29	0.7
	Moderate	1318, 31.87%, 30.45–33.29	1326, 32.06%, 30.64–33.48	
	None	437, 10.57%, 9.63–11.50	408, 9.86%, 8.96–10.77	
	Severe	757, 18.30%, 17.12–19.48	755, 18.25%, 17.08–19.43	
	Not reported	257, 6.21%, 5.48–6.95	247, 5.97%, 5.25–6.69	
Frequency of symptoms (1–7, > 7)	1	279, 6.75%, 5.98–7.51	276, 6.67%, 5.91–7.43	0.92
	2	309, 7.47%, 6.67–8.27	322, 7.79%, 6.97–8.60	
	3	307, 7.42%, 6.62–8.22	313, 7.57%, 6.76–8.37	
	4	253, 6.12%, 5.39–6.85	272, 6.58%, 5.82–7.33	
	5	289, 6.99%, 6.21–7.76	267, 6.46%, 5.71–7.20	
	6	271, 6.55%, 5.80–7.31	279, 6.75%, 5.98–7.51	
	7	1339, 32.37%, 30.95–33.80	1354, 32.74%, 31.31–34.17	
	< 1	669, 16.18%, 15.05–17.30	643, 15.55%, 14.44–16.65	
	Not reported	420, 10.15%, 9.23–11.08	410, 9.91%, 9.00–10.82	

Abbreviations: % = Percentage, CI = Confidence Interval, *n*: number of samples, UAS7 = Urticaria Activity Score (7‐day assessment)—evaluates the activity of CSU symptoms like wheals and itch, UCT = Urticaria Control Test—a score to measure disease control in Chronic Spontaneous Urticaria (CSU).

### 3.3 Synthetic Data of Patient Subgroups Accurately Reflects RWD

A subgroup of patients over 60 years old was analyzed to compare gender, BMI, number of comorbidities, UAS7, and UCT score distributions between synthetic and RWD, with no significant differences observed (*p* > 0.12 for all comparisons). Similar results were found for patients with BMI ≥ 25 and BMI ≥ 30, as well as for female and male subgroups, with no statistically significant differences in age, BMI, number of comorbidities, UAS7, or UCT scores (*p* > 0.10 for all; Appendix Table 2).

### 3.4 Synthetic Data Shows Similar Disease‐Relevant Correlations

To assess whether quantitative measures can be correlated with common correlation methods such as Pearson and Spearman, UCT scores were correlated with UAS7 scores using Spearman’s rho, revealing a strong negative correlation in both the RWD (−0.73) and the synthetic data (−0.7; *p* = 0.6 for the difference). Similarly, the correlation tested between age and disease duration for the RWD displayed a Pearson’s *r* of 0.2, whereas the synthetic data showed a Pearson’s *r* of 0.2 (*p* = 0.6 for the difference). The relationship between UAS7/UCT scores and age was examined using linear regression, while associations with gender were evaluated through logistic regression. For UCT and age, the beta coefficient in RWD reached 0.01 (95% CI: [0, 0.02]), and in synthetic data, 0.02 (95% CI: [0.01, 0.03]). For UAS7 and age, both RWD and synthetic data yielded a beta coefficient of −0.03 (RWD 95% CI: [−0.07, 0.01]; synthetic 95% CI: [−0.06, 0.01]). Logistic regression estimates for UCT and gender produced ORs of 1.02 (RWD 95% CI: [1.01, 1.04]) and 1.03 (synthetic 95% CI: [1.02, 1.05]). For UAS7 and gender, ORs were 0.99 (RWD 95% CI: [0.99, 1]) and 0.99 (synthetic 95% CI: [0.99, 1]). No significant differences emerged between RWD and synthetic datasets (Table 3).

**TABLE 3:** Bivariate associations compared between real world and synthetic data.

Association measures	Real‐world sata [*ρ*/beta coefficient and CI]	Synthetic data [rho/beta coefficient and CI]	*p* value interaction data × IV
UCT and UAS7 (Spearman correlation)	ρ: −0.731	*ρ*: −0.717	0.58
Age and disease duration (Pearson correlation)	*ρ*: 0.170	*ρ*: 0.153	0.558
UCT and age (linear regression)	Beta: 0.012 95% CI: [0.0015, 0.022]	Beta: 0.018 95% CI: [0.0078, 0.028]	0.42
UAS7 and age (linear regression)	Beta: −0.028 95% CI: [−0.066, 0.0091]	Beta: ‐ 0.028 95% CI: [−0.064, 0.0094]	0.98
UCT and gender (logistic regression)	OR: 1.02 95% CI: [1.01, 1.04]	OR: 1.03 95% CI: [1.02, 1.05]	0.44

Abbreviations: % = Percentage, Beta coefficient (β) = Measures the effect size, CI = Confidence Interval, *n* = number of samples, Rho (*ρ*) = Measures the strength and direction of the correlation, UAS7 = Urticaria Activity Score (7‐day assessment)—evaluates the activity of CSU symptoms like wheals and itch, UCT = Urticaria Control Test—a score to measure disease control in Chronic Spontaneous Urticaria (CSU).

### 3.5 A Patient Population of 25% Allows the Generation of a Four Times Larger Synthetic Population, While Maintaining High‐Quality Data

To operationalize the generation of synthetic data, we defined the objective of designing a hypothetical pilot study that is sufficiently powered. The goal was to identify the lowest threshold at which synthetic patients could be generated without significant differences from real patients.

To begin with, we calculated the minimal sample size needed for a 1:1 treatment/placebo design, an effect size of 0.6, and a 25% dropout rate (to achieve 80% power with an alpha of 5%). The estimated sample size resulted in a total of 150 patients (75 per arm), which is large considering the patients' participation/willingness from previous CSU trials. From the entire dataset, a random selection of 150 patients was made. Using this subset, synthetic data was generated at varying proportions (100%, 50%, 20%, and 10%). Next, a range of variables was compared between the generated synthetic data and the original (Table 4). In this experiment, the lowest threshold was found to be 25%, which means that enrolling just 38 patients in a clinical trial and applying GenDT allows us to generate a synthetic cohort of 150 patients. In other words, we can produce a synthetic patient population that is four times larger while maintaining high‐quality data. In this study, high‐fidelity synthetic datasets were produced using reduced sample sizes without modifying the number of covariates. While downsampling preserved data quality, the influence of covariate reduction remains untested. Theoretically, limiting covariates may compromise the ability to model complex relationships, potentially reducing the utility of the synthetic data for downstream analyses.

**TABLE 4:** Evaluating the potential reduction in sample size using synthetic data to power UAS7 assessment in chronic spontaneous urticaria (CSU) research.

Percentage of subset (*n* = 150)	Gender (Dichotomous; F vs. M)	Age (quantitative; scale; normal)	UAS7 (quantitative; ordinal; non normal)	UCT (quantitative; ordinal; normal)	Medications (multinomial; 5 levels)
100%	0.36	0.15	0.12	0.94	0.30
50% (*n* = 75)	0.1	0.74	0.46	0.39	0.81
25% (*n* = 38)	0.22	0.58	0.41	0.57	0.65
20% (*n* = 30)	0.53	0.88	0.05	0.69	0.0001
10% (*n* = 15)	0.71	0.04	0.056	0	0.00004

Abbreviations: CSU = Chronic Spontaneous Urticaria, F = Female, M = Male, UAS7 = Urticaria Activity Score (7‐day assessment), UCT = Urticaria Control Test.

## 4 Discussion

Our study demonstrated that synthetic data could effectively mimic RWD, with no significant differences observed across studied variables, including patient subgroups and variable associations. We demonstrate that two quantitative measures can be effectively replicated in synthetic data using common correlation methods, and that associations could be modeled accordingly. A sensitivity analysis revealed that generative model performance remained acceptable down to 25% of the initial dataset size. These findings align with other research [27] that found univariate distributions within 1% difference on an information‐theoretic metric as well as with findings which showed that synthetic data models can augment real data, up sample or rebalance datasets [9], and generate data for rare events or edge cases [9, 27].

We observed that multinomial and dichotomous variables, such as treatment modalities, were more prone to errors than quantitative variables, particularly when generated from small samples sizes. If data was scarce for single categories of an ordinal scale, we observed that generated data may not reflect the real data as accurately. Synthetic models often assume that populations are more homogeneous than they are. They may fail to replicate the full range of diversity (e.g., age, genetics, lifestyle, comorbidities). Nevertheless, while the role of missing values and non‐normal data distribution in affecting results requires further investigation, their presence did not affect the similarity between RWD and synthetic data; this is even true for quantitative variables arising from lower sample sizes.

CIs reflecting differences between parameters from both datasets were displayed, supplemented with *post hoc* analyses reporting CIs and *p*‐values. These metrics are widely accepted as standard measures for evaluating the quality and validity of synthetic data [28]. Based on these results, external testing on an entirely separate dataset and prospective validation within the context of ongoing clinical trials should be conducted. Expanding the dataset to include a larger and more diverse patient population will enhance the model’s reproducibility and accuracy, ensuring it generalizes well across various clinical scenarios [6].

Currently, there is a lack of specific artificial intelligence (AI) standards for synthetic data generation [29]^.^

While existing studies primarily report metrics like sensitivity, specificity, or accuracy [30], there is a growing need for more comprehensive evaluation methods. To further confirm non‐significance between real and synthetic data, researchers should determine the minimal acceptable difference between parameters before synthetic data generation, similar to non‐inferiority clinical trials [31]. Additionally, establishing acceptable CIs for these comparisons is crucial. One promising approach to address uncertainty in synthetic data evaluation is the Conformal Prediction (CP) [21]. This method provides measures of uncertainty and can help quantify the reliability of synthetic data generation models. Consequently, by incorporating CP alongside CI and significance values, we could develop and apply more robust standards for assessing the quality and utility of synthetic datasets in AI applications.

Furthermore, this work shows a 75% lower sample size for both control and treatment arms while achieving equivalent statistical power. Existing AI‐based digital twin technologies, such as Unlearn.AI, which are approved by the FDA and EMA for use in pivotal studies, achieve only a 33% reduction in control arm size. Further testing against other technologies is needed to benchmark performance and validate scalability. The FDA’s AI healthcare initiative underscores the value of synthetic data in simulating clinical scenarios, enhancing drug development pipelines, and supporting post‐market surveillance [32, 33]. This scalability addresses the critical need for large, diverse datasets to enable robust analyses and detect rare safety signals often missed in underpowered traditional studies.

## 5 Limitations

RWD is known to be less controlled than synthetic world data and may be imbalanced for some subgroups (small number of observations in the original variable from the RWD), so that the algorithm has a scarcity of data to learn from, and bias may be created if one characteristic is generated more frequently than another and does not identically reflect the underlying distribution as given in the RWD. Furthermore, the weaknesses that apply to RWD also apply to the synthetic data, including selection, information, and residual confounding biases. Further applications are necessary to assess the effect of every weakness on the outcomes and find ways to mitigate it.

## 6 Conclusion

Synthetic data could replicate RWD with reasonable accuracy for CSU patients; the potential of this method in extending small patient subgroup data is suggested for clinical and epidemiological research. A synthetic patient population that is four times larger while maintaining high‐quality data could be reproduced. Further research is necessary to establish and validate the standards of this method, allowing the scientific community to benefit from its advantages and safely use it in research settings.

## Author Contributions

**Annika Gutsche:** conceptualization, methodology, writing – original draft, validation, formal analysis, writing – review and editing, investigation, data curation, project administration. **Pascale Salameh:** conceptualization, writing – review and editing, formal analysis, supervision, methodology, data curation, validation. **Samad S. Jahandideh:** conceptualization, writing – review and editing, methodology, validation, visualization, formal analysis. **Mehran Roodsaz:** writing – review and editing, conceptualization, resources. **Serkan Kutan:** writing – review and editing, conceptualization, resources. **Ali Salehzadeh‐Yazdi:** writing – review and editing, methodology, validation. **Emek Kocatürk:** resources, writing – review and editing. **Stamatios Gregoriou:** writing – review and editing, resources. **Simon F. Thomsen:** writing – review and editing, resources. **Kanokvalai Kulthanan:** resources, writing – review and editing. **Papapit Tuchinda:** writing – review and editing, resources. **Joachim Dissemond:** resources, writing – review and editing. **Alicja Kasperska‐Zajac:** writing – review and editing, resources. **Magdalena Zajac:** resources, writing – review and editing. **Mateusz Zamłyński:** writing – review and editing, resources. **Martijn van Doorn:** resources, writing – review and editing. **Claudio A. S. Parisi:** writing – review and editing, resources. **Jonny G. Peter:** writing – review and editing, resources. **Cascia Day:** resources, writing – review and editing. **Cathryn McDougall:** writing – review and editing, resources. **Michael Makris:** writing – review and editing, resources. **Daria Fomina:** writing – review and editing, resources. **Elena Kovalkova:** resources, writing – review and editing. **Nikolai Streliaev:** writing – review and editing, resources. **Gerelma Andrenova:** resources, writing – review and editing. **Marina Lebedkina:** resources, writing – review and editing. **Maryam Khoskhkui:** resources, writing – review and editing. **Mehraneh M. Aliabadi:** resources, writing – review and editing. **Andrea Bauer:** resources, writing – review and editing. **Lea Kiefer:** conceptualization, resources, writing – review and editing. **Melba Munoz:** conceptualization, resources, writing – review and editing. **Karsten Weller:** resources, writing – review and editing. **Pavel Kolkhir:** conceptualization, resources, writing – review and editing. **Martin Metz:** supervision, conceptualization, writing – review and editing, resources, project administration.

## Conflicts of Interest

A. Gutsche, P. Salameh, A. Salehzadeh‐Yazdi, A. Kasperska‐Zajac, M. Zajac, M. Zamłyński, C. A. S. Parisi, C. McDougall, E. Kovalkova, N. Streliaev, G. Andrenova, M. M. Aliabadi, and L. Kiefer declare no conflicts of interest. S. Jahandideh reports no conflict of interest, he is a cofounder of Tediax B.V. which is a startup that develops GenAI algorithms. M. Roodsaz and S. Kutan reports no conflict of interest and also a cofounder of Tediax. E. Kocatürk has received grants for contracts from Almirall. S. Gregoriou has received Consulting fees and Payment or honoraria for lectures, presentations, speakers' bureaus, manuscript writing or educational events as well as support for attending meetings and/or travel from Novartis, AbbVie, Pfizer, Lilly. S. Thomsen has received Grants or contracts from Sanofi, AbbVie, LEO Pharma, Novartis, UCB Pharma, Janssen Pharmaceuticals, Almirall and Payment or honoraria for lectures, presentations, speakers bureaus, manuscript writing or educational events Sanofi, AbbVie, LEO Pharma, Novartis, UCB Pharma, Janssen, Pharmaceuticals, Almirall and Participation on a Data Safety Monitoring Board or Advisory Board from Sanofi, AbbVie, LEO Pharma, Pfizer, Eli Lilly, Novartis, UCB Pharma, Almirall, Union Therapeutics, Symphogen, Janssen Pharmaceuticals as well as Leadership or fiduciary role in other board, society, committee or advocacy group, paid or unpaid from Danish Dermatological Society. K. Kulthanan has received educational speaker fees from Novartis, Menarini, Sanofi Genzyme. P. Tuchinda has received educational speaker fees from Sanofi Genzyme. J. Dissemon has received Participation on a Data Safety Monitoring Board or Advisory Board. M. van Doorn received grants or contracts from Janssen, Almirall, ThirdHarmonic, Escient as well as payment or honoraria for lectures, presentations, speakers' bureaus, manuscript writing or educational events from Novartis, AbbVie, LEO pharma, Sanofi, Janssen, UCB, BMS and Support for attending meetings and/or travel from USB. J. Peter has received grants from Takeda, Kalvista, Astria, Pharvaris as well as honoraria as a speaker and/or advisor for CSL Behring, Takeda, Novartis, Sanofi Regeneron, support for attending meeting and/or travel from Sanofi, HAE international and support for Participation on a Data Safety Monitoring Board or Advisory Board from Pharvaris and Astria. C. Day received grants or contracts from Discovery Foundation Academic Award for PhD and the South African Medical Research Council Division of Research Capacity Development, Clinician Researcher Programme as well as payment or honoraria for lectures, presentations, speakers' bureaus, manuscript writing or educational events from Taskeda, The Allergy Foundation of South Africa (AFSA) and The Allergy Society of South Africa. She got support for attending meetings and/or travel for the C1 inhibitor and Angioedema Workshop and holds a Leadership or fiduciary role in other board, society, committee or advocacy group, paid or unpaid in the Allergy Society of South Africa Executive Committee. M. Makris has received payment or honoraria for lectures, presentations, speakers' bureaus, manuscript writing or educational events from Astra Zeneca, Prizer, Gsk, Elpen, Menarini. D. Fomina participates on a Data Safety Monitoring Board or Advisory Board for CURE ISC and holds a leadership or fiduciary role for European Academy of Allergy and Clinical Immunology (EAACI), GA2LEN Urticaria Center of Reference and Excellence (UCARE), GA2LEN Atopic Dermatitis Centers of Reference and Excellence (ADCARE). M. Lebedkina holds leadership or fiduciary role for EAACI. M. Khoskhkui is or recently was a speaker and/or advisor for and/or has received research funding from Abidi Pharma, Alhavi Pharma, AstraZeneca, Actover, Ofogh Tolid Darou pars, Kimia salamat nikan, CinnaGen, Sanofi, GlaxoSmithKline and Danon, outside of submitted work. A. Bauer receive consulting fees from Novartis, Sanofi, Otsuka, Kalvista, Leo as well as Payment or honoraria for lectures, presentations, speakers' bureaus, manuscript writing or educational events from Novartis, Sanofi, CSL Behring, Leo, Almirall, Biocyst, L Oreal. She got support for attending meetings and/or travel from Sanofi, Leo, Novartis, CSL Behring, Otsuka, Kalvita and holds a leadership or fiduciary role in other board, society, committee or advocacy group, paid or unpaid for the Working Group for Occupational and Environmental Dermatology as well as the German Contact Dermatitis Research Group. She has other financial or non‐financial interests regarding clinical studies for Abbvie, Escient, Galderma, Jasper, Celldex, Amgen, AstraZeneca, Novartis, Sanofi, Genentech, Janssen, Pierre Fabre, Gilead, Incyte, Leo, Almirall, Lilly, Pfizer, Regeneron. M. Munoz has received Grants or contracts from Celltrion, Jasper, Biocryst, Annexon, Roche and Consulting fees from Biocryst, Jasper as well as Payment or honoraria for lectures, presentations, speakers' bureaus, manuscript writing or educational events from Celltrion and Support for attending meetings and/or travel from Celldex, Galen. K. Weller is or recently was a speaker and/or advisor for and/or has received research funds from Moxie, Novartis, Sanofi, Takeda, and Noucor (Uriach). P. Kolkhir has received honoraria as a speaker and/or advisor for: BioCryst, Merus, Novartis, Roche and ValenzaBio, outside of the submitted work. M. Metz has received honoraria as a speaker and/or advisor for: AbbVie, Advanz, ALK‐Abello, Allegria, Almirall, Amgen, Argenx, AstraZeneca, Astria, Attovia, Berlin‐Chemie, Celldex, Celltrion, DeepApple, Escient, Galderma, GSK, Incyte, Jasper, Lilly, Novartis, Pfizer, Regeneron, Sanofi, Santa Ana Bio, Septerna, Teva, ThirdHarmonicBio, and Vifor.

## Supporting information

Table S1

## Data Availability

The data that support the findings of this study are available from the corresponding author upon reasonable request.

## Appendix A

**TABLE A1:** Proportions of specific comorbidities compared between real‐world and synthetic data.

Dataset		*n*	Percent (%)	95% CI (lower and upper limits)		*p* value
Atopic dermatitis						
Original	No	3683	89	88.1	90	0.9472
Original	Yes	197	4.76	4.11	5.41	
Original	Not reported	256	6.19	5.46	6.92	
Synthetic	No	3694	89.3	88.4	90.3	
Synthetic	Yes	200	4.84	4.18	5.49	
Synthetic	Not reported	242	5.85	5.14	6.57	
Allergic Rhinitis						
Original	No	3091	74.7	73.4	76.1	0.9802
Original	Yes	790	19.1	17.9	20.3	
Original	Not reported	255	6.17	5.43	6.9	
Synthetic	No	3112	75.2	73.9	76.6	
Synthetic	Yes	793	19.2	18	20.4	
Synthetic	Not reported	231	5.59	4.89	6.28	
Asthma						
Original	No	3452	83.5	82.3	84.6	0.7434
Original	Yes	442	10.7	9.75	11.6	
Original	Not reported	242	5.85	5.14	6.57	
Synthetic	No	3471	83.9	82.8	85	
Synthetic	Yes	433	10.5	9.54	11.4	
Synthetic	Not reported	232	5.61	4.91	6.31	
NSAID hypersensitivity						
Original	No	3510	84.9	83.8	86	0.808
Original	Yes	266	6.43	5.68	7.18	
Original	Not reported	360	8.7	7.84	9.56	
Synthetic	No	3485	84.3	83.2	85.4	
Synthetic	Yes	271	6.55	5.8	7.31	
Synthetic	Not reported	380	9.19	8.31	10.1	
Thyroid disease						
Original	No	2929	70.8	69.4	72.2	0.8165
Original	Yes	655	15.8	14.7	16.9	
Original	Not reported	552	13.3	12.3	14.4	
Synthetic	No	2900	70.1	68.7	71.5	
Synthetic	Yes	659	15.9	14.8	17	
Synthetic	Not reported	577	14	12.9	15	
Autoimmune diseases						
Original	No	3304	79.9	78.7	81.1	0.9378
Original	Yes	413	9.99	9.07	10.9	
Original	Not reported	419	10.1	9.21	11.1	
Synthetic	No	3292	79.6	78.4	80.8	
Synthetic	Yes	415	10	9.12	10.9	
Synthetic	Not reported	429	10.4	9.44	11.3	
Diabetes mellitus						
Original	No	3540	85.6	84.5	86.7	0.9952
Original	Yes	238	5.75	5.04	6.46	
Original	Not reported	358	8.66	7.8	9.51	
Synthetic	No	3537	85.5	84.4	86.6	
Synthetic	Yes	239	5.78	5.07	6.49	
Synthetic	Not reported	360	8.7	7.84	9.56	
Celiac disease						
Original	No	3741	90.4	89.6	91.3	0,8112
Original	Yes	10	0.242	0.0921	0.391	
Original	Not reported	385	9.31	8.42	10.2	
Synthetic	No	3748	90.6	89.7	91.5	
Synthetic	Yes	8	0.193	0,0595	0.327	
Synthetic	Not reported	380	9.19	8.31	10.1	
Hypertension						
Original	No	3037	73.4	72.1	74.8	0.9593
Original	Yes	775	18.7	17.5	19.9	
Original	Not reported	324	7.83	7.01	8.65	
Synthetic	No	3035	73.4	72	74.7	
Synthetic	Yes	778	18.8	17.6	20	
Synthetic	Not reported	323	7.81	6.99	8.63	
Hyperlipidemia						
Original	No	3225	78	76.7	79.2	0.7206
Original	Yes	452	10.9	9.98	11.9	
Original	Not reported	459	11.1	10.1	12.1	
Synthetic	No	3227	78	76.8	79.3	
Synthetic	Yes	465	11.2	10.3	12.2	
Synthetic	Not reported	444	10.7	9.79	11.7	
Obesity						
Original	No	3283	79.4	78.1	80.6	0.7966
Original	Yes	543	13.1	12.1	14.2	
Original	Not reported	310	7.5	6.69	8.3	
Synthetic	No	3297	79.7	78.5	80.9	
Synthetic	Yes	535	12.9	11.9	14	
Synthetic	Not reported	304	7.35	6.55	8.15	
Metabolic syndrome						
Original	No	3675	88.9	87.9	89.8	0.9689
Original	Yes	93	2.25	1.8	2.7	
Original	Not reported	368	8.9	8.03	9.77	
Synthetic	No	3692	89.3	88.3	90.2	
Synthetic	Yes	95	2.3	1.84	2.75	
Synthetic	Not reported	349	8.44	7.59	9.29	
Myeloproliferative disease						
Original	No	3738	90.4	89.5	91.3	0.7966
Original	Yes	9	0.218	0.0756	0.36	
Original	Not reported	389	9.41	8.52	10.3	
Synthetic	No	3754	90.8	89.9	91.6	
Synthetic	Yes	7	0.169	0.044	0.295	
Synthetic	Not reported	375	9.07	8.19	9.94	
Gasterointestinal disease						
Original	No	3037	73.4	72.1	74.8	0.6478
Original	Yes	781	18.9	17.7	20.1	
Original	Not reported	318	7.69	6.88	8.5	
Synthetic	No	3078	74.4	73.1	75.7	
Synthetic	Yes	770	18.6	17.4	19.8	
Synthetic	Not reported	288	6.96	6.19	7.74	
Depression						
Original	No	3516	85	83.9	86.1	0.7439
Original	Yes	299	7.23	6.44	8.02	
Original	Not reported	321	7.76	6.95	8.58	
Synthetic	No	3522	85.2	84.1	86.2	
Synthetic	Yes	309	7.47	6.67	8.27	
Synthetic	Not reported	305	7.37	6.58	8.17	
Anxiety						
Original	No	3383	81.8	80.6	83	0.8257
Original	Yes	444	10.7	9.79	11.7	
Original	Not reported	309	7.47	6.67	8.27	
Synthetic	No	3399	82.2	81	83.3	
Synthetic	Yes	438	10.6	9.65	11.5	
Synthetic	Not reported	299	7.23	6.44	8.02	
Toxic habit						
Original	No	3161	76.4	75.1	77.7	0.6438
Original	Yes	343	8.29	7.45	9.13	
Original	Not reported	632	15.3	14.2	16.4	
Synthetic	No	3152	76.2	74.9	77.5	
Synthetic	Yes	356	8.61	7.75	9.46	
Synthetic	Not reported	628	15.2	14.1	16.3	
Food allergy						
Original	No	3451	83.4	82.3	84.6	0.5601
Original	Yes	173	4.18	3.57	4.79	
Original	Not reported	512	12.4	11.4	13.4	
Synthetic	No	3471	83.9	82.8	85	
Synthetic	Yes	162	3.92	3.33	4.51	
Synthetic	Not reported	503	12.2	11.2	13.2	

Abbreviations: % = Percentage, CI = Confidence Interval, *n* = number of samples.

**TABLE A2:** Comparison of demographic and clinical parameters in subgroups (e.g. elderly, higher/lower BMI, female/male) between real‐world and synthetic data.

Sub‐population	Dataset	Characteristics	*n*/mean	Percent (%)/SD	*p* value
Elderly > 60 yrs					
		Gender			0.64
	Original	Female	564	73.2	
	Original	Male	206	26.8	
	Synthetic	Female	555	72.1	
	Synthetic	Male	215	27.9	
		UCT			0.52
	Original		8.71	4.91	
	Synthetic		8.91	5.03	
		UAS7			0.93
	Original		15.61	13.37	
	Synthetic		15.52	13.32	
		Number of comorbidities			0.12
	Original		2.74	1.93	
	Synthetic		2.59	1.76	
		BMI			0.76
	Original		28.23	8.64	
	Synthetic		28.39	8.58	
BMI ≥ 25 overweight					
		Age			0.29
	Original		48.3	14.6	
	Synthetic		48.8	14.8	
		Gender			0.91
	Original	Female	1277	69.7	
	Original	Male	555	30.3	
	Synthetic	Female	1273	69.5	
	Synthetic	Male	559	30.5	
		UCT			0.53
	Original		8.14	4.73	
	Synthetic		8.25	4.77	
		UAS7			0.61
	Original		17.4	13.84	
	Synthetic		17.1	13.87	
		Number of comorbidities			0.65
	Original		2.53	2	
	Synthetic		2.5	1.9	
		BMI			0.59
	Original		30.32	6.81	
	Synthetic		30.46	8.24	
BMI ≥ 30 obese					
		Age			0.21
	Original		49.9	13.8	
	Synthetic		49	14	
		Gender			0.13
	Original	Female	572	75.8	
	Original	Male	183	24.2	
	Synthetic	Female	545	72.2	
	Synthetic	Male	210	27.8	
		UCT			0.2
	Original		7.95	4.82	
	Synthetic		8.3	4.73	
		UAS7			0.1
	Original		17.58	13.71	
	Synthetic		16.02	13.3	
		Number of comorbidities			0.15
	Original		3.22	2.15	
	Synthetic		3.06	2.02	
		BMI			0.52
	Original		34.83	8.67	
	Synthetic		35.11	8.11	
Female					
		Age			0.59
	Original		44.6	16	
	Synthetic		44.8	16	
		UCT			0.93
	Original		8.1	4.61	
	Synthetic		8.11	4.68	
		UAS7			0.24
	Original		17.4	13.57	
	Synthetic		16.82	13.67	
		Number of comorbidities			0.43
	Original		2.08	1.8	
	Synthetic		2.04	1.68	
		BMI			0.51
	Original		26.16	6.5	
	Synthetic		26.04	6.42	
Male					
		Age			0.88
	Original		43.3	16.8	
	Synthetic		43.2	16.4	
		UCT			0.88
	Original		8.62	4.66	
	Synthetic		8.65	4.51	
		UAS7			0.7
	Original		16.7	13.81	
	Synthetic		16.4	13.64	
		Number of comorbidities			0.35
	Original		1.72	1.58	
	Synthetic		1.66	1.54	
		BMI			0.5
	Original		26.56	7.2	
	Synthetic		26.78	7.1	

Abbreviations: % = Percentage, BMI = Body Mass Index, CI = Confidence Interval, F = Female, M = Male, *n* = number of samples, UAS7 = Urticaria Activity Score (7‐day assessment)—evaluates the activity of CSU symptoms like wheals and itch, UCT = Urticaria Control Test—a score to measure disease control in Chronic Spontaneous Urticaria (CSU), yrs = Years.

## References

[1] N. Chaudhari , R. Ravi , N. Gogtay , and U. Thatte , “Recruitment and Retention of the Participants in Clinical Trials: Challenges and Solutions,” Perspectives in Clinical Research 11, no. 2 (2020): 64–69, 10.4103/picr.PICR_206_19. 32670830 PMC7342338

[2] J. Fricke , G. Ávila , T. Keller , et al., “Prevalence of Chronic Urticaria in Children and Adults Across the Globe: Systematic Review With Meta‐Analysis,” Allergy 75, no. 2 (2020): 423–432, 10.1111/all.14037. 31494963

[3] M. Hutson , “How AI Is Being Used to Accelerate Clinical Trials,” Nature 627, no. 8003 (2024): S2–S5, 10.1038/d41586-024-00753-x. 38480968

[4] N. Papapostolou , P. Xepapadaki , A. Katoulis , and M. Makris , “Comorbidities of Chronic Urticaria: A Glimpse Into a Complex Relationship,” Frontiers in Allergy 3 (2022): 1008145, 10.3389/falgy.2022.1008145. 36465885 PMC9712803

[5] C. Patruno , G. Fabbrocini , F. Cillo , G. Torta , L. Stingeni , and M. Napolitano , “Chronic Urticaria in Older Adults: Treatment Considerations,” Drugs & Aging 40, no. 3 (2023): 165–177, 10.1007/s40266-023-01010-y. 36808569

[6] J. Chen , D. Chun , M. Patel , E. Chiang , and J. James , “The Validity of Synthetic Clinical Data: A Validation Study of a Leading Synthetic Data Generator (Synthea) Using Clinical Quality Measures,” BMC Medical Informatics and Decision Making 19, no. 1 (2019): 44, 10.1186/s12911-019-0793-0. 30871520 PMC6416981

[7] P. Kolkhir , H. Bonnekoh , M. Metz , and M. Maurer , “Chronic Spontaneous Urticaria: A Review,” JAMA 332, no. 17 (September 2024): 1464, 10.1001/jama.2024.15568. 39325444

[8] F. Liu and D. Panagiotakos , “Real‐World Data: A Brief Review of the Methods, Applications, Challenges and Opportunities,” BMC Medical Research Methodology 22, no. 1 (2022): 287, 10.1186/s12874-022-01768-6. 36335315 PMC9636688

[9] L. Kühnel , J. Schneider , I. Perrar , et al., “Synthetic Data Generation for a Longitudinal Cohort Study–Evaluation, Method Extension and Reproduction of Published Data Analysis Results,” Scientific Reports 14, no. 1 (2024): 14412, 10.1038/s41598-024-62102-2. 38909025 PMC11193715

[10] K. El Emam , L. Mosquera , and J. Bass , “Evaluating Identity Disclosure Risk in Fully Synthetic Health Data: Model Development and Validation,” Journal of Medical Internet Research 22, no. 11 (2020): e23139, 10.2196/23139. 33196453 PMC7704280

[11] A. Guo , R. E. Foraker , R. M. MacGregor , F. M. Masood , B. P. Cupps , and M. K. Pasque , “The Use of Synthetic Electronic Health Record Data and Deep Learning to Improve Timing of High‐Risk Heart Failure Surgical Intervention by Predicting Proximity to Catastrophic Decompensation,” Frontiers in Digital Health 2 (2020): 576945, 10.3389/fdgth.2020.576945. 34713050 PMC8521851

[12] A. Gonzales , G. Guruswamy , and S. R. Smith , “Synthetic Data in Health Care: A Narrative Review,” PLOS Digital Health 2, no. 1 (2023): e0000082, 10.1371/journal.pdig.0000082. 36812604 PMC9931305

[13] B. Nowok , G. M. Raab , and C. Dibben , “Providing Bespoke Synthetic Data for the UK Longitudinal Studies and Other Sensitive Data With the Synthpop Package for R 1,” Statistical Journal of the IAOS 33, no. 3 (2017): 785–796, 10.3233/sji-150153.

[14] L. Mosquera , K. El Emam , L. Ding , et al., “A Method for Generating Synthetic Longitudinal Health Data,” BMC Medical Research Methodology 23, no. 1 (2023): 67, 10.1186/s12874-023-01869-w. 36959532 PMC10034254

[15] M. Giuffrè and D. L. Shung , “Harnessing the Power of Synthetic Data in Healthcare: Innovation, Application, and Privacy,” npj Digital Medicine 6, no. 1 (2023): 186, 10.1038/s41746-023-00927-3. 37813960 PMC10562365

[16] C. Xiao , E. Choi , and J. Sun , “Opportunities and Challenges in Developing Deep Learning Models Using Electronic Health Records Data: A Systematic Review,” Journal of the American Medical Informatics Association 25, no. 10 (2018): 1419–1428, 10.1093/jamia/ocy068. 29893864 PMC6188527

[17] T. Kokosi and K. Harron , “Synthetic Data in Medical Research,” BMJ Medicine 1, no. 1 (2022): e000167, 10.1136/bmjmed-2022-000167. 36936569 PMC9951365

[18] M. Sánchez‐Borges , I. J. Ansotegui , I. Baiardini , et al., “The Challenges of Chronic Urticaria Part 1: Epidemiology, Immunopathogenesis, Comorbidities, Quality of Life, and Management,” World Allergy Organization Journal 14, no. 6 (2021): 100533, 10.1016/j.waojou.2021.100533. 34221215 PMC8233382

[19] T. Therneau and B. Atkinson , “rpart: Recursive Partitioning and Regression Trees,” CRAN: Contributed Packages (April 1999), 10.32614/CRAN.package.rpart.

[20] W. Loh , “Fifty Years of Classification and Regression Trees,” International Statistical Review 82, no. 3 (2014): 329–348, 10.1111/insr.12016. PMC4380222 25844011

[21] L. Breiman , J. H. Friedman , R. A. Olshen , and C. J. Stone , Classification and Regression Trees, 1st ed. (Routledge, 2017). 10.1201/9781315139470.

[22] G. M. Raab , B. Nowok , and C. Dibben , “Practical Data Synthesis for Large Samples,” Journal of Privacy and Confidentiality 7, no. 3 (2018): 67–97, 10.29012/jpc.v7i3.407.

[23] B. Nowok , G. M. Raab , and C. Dibben , “synthpop: Bespoke Creation of Synthetic Data in R,” Journal of Statistical Software 74, no. 11 (2016): 1–26, 10.18637/jss.v074.i11.

[24] B. Ripley , “Tree: Classification and Regression Trees,” (December 2024), https://cran.r‐project.org/web/packages/tree/index.html.

[25] H. Wickham and M. H. Wickham , “Package Tidyverse. Easily Install Load ‘Tidyverse’,” (2017), http://r.meteo.uni.wroc.pl/web/packages/tidyverse/tidyverse.pdf.

[26] R. V. Lenth , B. Banfai , B. Bolker , et al., “emmeans: Estimated Marginal Means, Aka Least‐Squares Means,” (May 2025), https://cran.r‐project.org/web/packages/emmeans/index.html.

[27] Z. Azizi , C. Zheng , L. Mosquera , L. Pilote , and K. El Emam , “Can Synthetic Data Be a Proxy for Real Clinical Trial Data? A Validation Study,” BMJ Open 11, no. 4 (2021): e043497, 10.1136/bmjopen-2020-043497. PMC8055130 33863713

[28] R. E. Foraker , S. C. Yu , A. Gupta , et al., “Spot the Difference: Comparing Results of Analyses From Real Patient Data and Synthetic Derivatives,” JAMIA Open 3, no. 4 (2021): 557–566, 10.1093/jamiaopen/ooaa060. PMC7886551 33623891

[29] K2view , “Complete Handbook on Synthetic Data Generation | K2view,” accessed, October 8, 2024, https://www.k2view.com/what‐is‐synthetic‐data‐generation/.

[30] E. Esoimeme , “Examining The Potential Misuse of Artificial Intelligence to Circumvent Technology‐Based Processes For AML/CFT Compliance in The Cryptocurrency Ecosysyem,” CFT Compliance in The Cryptocurrency Ecosysyem (September 2024).

[31] G. Piaggio , D. R. Elbourne , S. J. Pocock , S. J. Evans , D. G. Altman , and for the CONSORT Group , “Reporting of Noninferiority and Equivalence Randomized Trials: Extension of the CONSORT 2010 Statement,” JAMA 308, no. 24 (2012): 2594–2604. 23268518 10.1001/jama.2012.87802

[32] B. Jiang , J. Zhang , Y. Jiang , and L. Kong , “FDA: Generating Fair Synthetic Data With Provable Trade‐Off Between Fairness and Faithfulness,” (October 2024), https://openreview.net/forum?id=UhdmcuuvSt.

[33] FDA , “Addressing the Limitations of Medical Data in AI,” (October 2024), https://www.fda.gov/medical‐devices/medical‐device‐regulatory‐science‐research‐programs‐conducted‐osel/addressing‐limitations‐medical‐data‐ai.

