# Supplementary material for: Can Synthetic Data Allow for Smaller Sample Sizes in Chronic Urticaria Research?
Source: Clin Transl Allergy. 2025 Aug 7;15(8):e70087. doi: 10.1002/clt2.70087 (PMC12329239; doi:10.1002/clt2.70087)
Supplement: Supplementary file 1 — Table S1 [file CLT2-15-e70087-s001.docx]

**SUPPLEMENTARY MATERIAL**

**Supplementary Table 1. List of centers participating in this study***. Frequencies and percentages of the included patients of each center in the study are shown.*

| **CURE Center** | **Frequency** | **Percent (%)** |
| --- | --- | --- |
| DE \| Allergie-Centrum-Charite | 644 | 15.6 |
| RU \| Moscow \| CSH 52 | 634 | 15.3 |
| PL \| Zabrze | 398 | 9.6 |
| ES \| Barcelona | 209 | 5.1 |
| TH \| Bangkok | 169 | 4.1 |
| IR \| Mashhad | 151 | 3.7 |
| ZA \| Cape Town | 128 | 3.1 |
| SI \| Golnik | 106 | 2.6 |
| FR \| Montpellier | 101 | 2.4 |
| GR \| Athen-Andreas Sygros | 93 | 2.2 |
| RU \| Smolensk | 90 | 2.2 |
| AE \| Abu Dhabi | 89 | 2.2 |
| FR \| CHU Grenoble | 88 | 2.1 |
| GR \| Athen | 88 | 2.1 |
| DK \| Copenhagen | 81 | 2 |
| DE \| UMC-Mainz | 74 | 1.8 |
| AR \| Buenos Aires | 72 | 1.7 |
| DE \| UK-Essen | 71 | 1.7 |
| DE \| Uniklinikum Dresden | 71 | 1.7 |
| RU \| Moscow | 69 | 1.7 |
| NL \| Rotterdam | 63 | 1.5 |
| IT \| Milan | 50 | 1.2 |
| CN \| Suzhou | 48 | 1.2 |
| TR \| Koç University Hospital | 46 | 1.1 |
| TR \| Sakarya | 43 | 1 |
| JP \| Yokohama | 34 | 0.8 |
| CO \| Medellín | 34 | 0.8 |
| BR \| Salvador-BA | 31 | 0.7 |
| DE \| Uniklinikum Jena | 30 | 0.7 |
| PL \| Lublin | 30 | 0.7 |
| TR \| Pendik Istanbul | 27 | 0.7 |
| JP \| Hiroshima | 26 | 0.6 |
| BR \| São Paulo | 25 | 0.6 |
| FR-Paris (aphp) | 24 | 0.6 |
| IR \| Teheran | 22 | 0.5 |
| IND \| Navi Mumbai | 17 | 0.4 |
| RU \| Kazan | 16 | 0.4 |
| FR \| Metz | 15 | 0.4 |
| CN \| Guangzhou | 14 | 0.3 |
| PT \| Porto | 14 | 0.3 |
| HR \| Zagreb | 13 | 0.3 |
| GR \| NKUA-Athen | 13 | 0.3 |
| PL \| Poznan | 11 | 0.3 |
| TR \| Istanbul | 9 | 0.2 |
| JP \| Kobe | 8 | 0.2 |
| DE \| Elbe Kliniken Buxtehude | 7 | 0.2 |
| IE \| St. James's Hospital | 7 | 0.2 |
| DE \| Justus-Liebig-Uni. Gießen | 6 | 0.1 |
| AR \| Bahía Blanca | 6 | 0.1 |
| EC \| Guayaquil | 3 | 0.1 |
| PT \| Coimbra (1) | 3 | 0.1 |
| BG \| Sofia | 3 | 0.1 |
| MX \| Hospital Espanol | 3 | 0.1 |
| PT \| Coimbra (2) | 3 | 0.1 |
| MK \| Skopje | 2 | 0 |
| SA \| King Khalid Hospital | 2 | 0 |
| TR \| Kayseri | 1 | 0 |
| IND \| Bengaluru | 1 | 0 |
| **Total** | 3492 | 100 |
